# Evaluation of carrot (*Daucus carota L.)* varieties for growth and yield as affected by NPSB fertilizer rates in Gondar district, Ethiopia

**DOI:** 10.3389/fpls.2025.1505302

**Published:** 2025-01-31

**Authors:** Abebaw Mulugeta, Fentahun Asrat, Derajew Asres, Shiferaw Mebrat

**Affiliations:** ^1^ Department of Horticulture, College of Agriculture and Environmental Sciences, University of Gondar, Gondar, Ethiopia; ^2^ Department of Horticulture, College of Agriculture, Food and Climate Sciences, Injibara University, Injibara, Ethiopia; ^3^ Department of Plant Sciences, College of Agriculture and Environmental Sciences, University of Gondar, Gondar, Ethiopia

**Keywords:** blended fertilizer, growth, varieties, planting year, root yield

## Abstract

Carrot (*Daucus carota* L.) is one of the most important root crops grown worldwide and in Ethiopia. However, its production and productivity are low due to a lack of improved varieties and unbalanced fertilizer rates, among other factors. The field experiment was, therefore, conducted to determine the performance of carrot varieties through blended fertilizer rates at Gondar district for a consecutive period of two years. The treatment consisted of six rates of blended NPSB (Nitrogen, phosphorus, sulfur and Boron) fertilizer (0, 40.6, 81.3, 122, 162.3, and 203.4 kg ha^-1^) and two carrot varieties (Haramay-I and Nantes), which were laid out in a randomized complete block design with three replications. The main effect of blended NPSB received in 162.3 kg ha^-1^ obtained the highest root diameter (3.38 cm), root length (20.93 cm), and root volume (110.60 mm). The main effect of the year was also the maximum number of leaves (10.3), root diameter (2.96 cm), root length (20.09 cm), and root volume (89.20 mm) recorded from the 2021 planting year. On the other hand, in the interaction of variety and NPSB, the highest root fresh weight (134.48 g plant^-1^) was obtained from the Haramaya-I variety and the application of 162.3 NPSB kg ha^-1^, while the lowest (57.13 g plant^-1^) was recorded by the Nantes variety with control. The highest dry matter (13.67%), marketable (50.77 t ha^-1^) and total (55.32 t ha^-1^) root yields were recorded from the interaction of 162.3 kg NPSB ha^-1^ and Haramaya-I variety. Therefore, the planting season and varietal selection should be considered in the carrot production area.

## 1 Introduction

Carrot (*Daucus carota* L.) belongs to the family Apiaceae, formerly Umbelliferae. Its root is valued as a food, mainly because of its high carotene content. Carrot is a biennial in the case of seed production, but it grows as an annual crop for root production. It belongs to the moderately hardy group of plants that are not particularly sensitive to winter cold or frost. It performs best under cool conditions, and its seeds germinate quite well (De Lannoy, 2001).

Carrot is an important vegetable crop that is widely used by humans, especially in children’s diets, owing to its high nutritional value (Heinonen, 1990). It is an important source of alpha- and beta-carotene, which are precursors of vitamin A, in human nutrition in many countries worldwide. They are weight loss-friendly foods that have been linked to lower cholesterol levels and improved eye health. Moreover, their antioxidant activity has been linked to reduced risk of cancer (Sharma et al., 2012).

Carrots are one of the major contributors to the world vegetable trade and are utilized in the fresh market and processing industries. The global annual production of carrots and turnips is estimated to be 44,762,859 tons (t), with a total production area of approximately 1,128,695 ha of land. China is the largest producer of carrot and turnip in the world, with an annual production of 2,148,297 tons on 455,656 ha of land. The total production volume in Africa is 236,301 tons, with the largest area, and the highest production is found in Nigeria and South Africa (FAO, 2019). According to the Ethiopian Central Statistical Agency (2020), the total production of carrots during the 2019/2020 cropping season in the country was 182,254.14 tons, with a total land area coverage of 4,073.63 ha.

In the Amhara region, the total production of carrot root was 3,578.10 tons, which was produced from 643.87 ha of land (CSA, 2020). In the study area of the Central Gondar zone, carrot production was estimated to be 35 tons, with a total area coverage of 12 ha (CGAO, 2021). The root yield of the Nantes carrot variety in Ethiopia ranges from 6 t ha^−1^ to 15 t ha^−1^ under farmer management, depending on agro-ecology (Tabor and Yesuf, 2012).

Application of NPK fertilizer at the recommended rates ensures successful production of carrot yields. The Haramaya-I variety was developed together with the Nantes variety by applying 100 kg DAP and 100 kg urea in a split application, which are recommended as a source of phosphorus and nitrogen, respectively, for root production (Wassu et al., 2014). N, P, and S play significant roles in the yield of carrots by enhancing photosynthesis and growth (Hart and Butler, 2003).

Carrot is among the top 10 most economically important vegetable crops in Ethiopia, yet its average productivity in the Amhara region is low at 5.5 t ha^−1^, significantly below the global average of 39 t ha^−1^ (CSA, 2020; FAO, 2019). Factors contributing to this low productivity include a shortage of improved varieties, lack of recommended fertilizer rates, low soil fertility, and pest and disease infestations (Alemayehu et al., 2015).

In the central Gondar zone, available phosphorous, available sulfur, and extractable boron are very low, whereas total nitrogen is low in the soil. Similarly, the soils of Lay Teda Kebele, where the study was conducted, contain very low available phosphorous, sulfur, extractable boron, and low total nitrogen (ATA, 2016). There was no shortage of extractable zinc in the study area. Hence, it is imperative to use blended NPSB fertilizers to increase the productivity of carrots in the study area. Ethiopian Agricultural Transformation Agency has also confirmed that N:P2O5:S:B (18.9N–37.7P2O5–6.95S–0.1B) fertilizer substituted DAP in all over part of crop growing area including carrot in Ethiopia. However, the response of carrot to the application rate of the newly introduced blended NPSB fertilizer under agro-ecological conditions in the central Gondar zone has not yet been studied.

In the Central Gondar Zone, particularly in the Gondar Zuria district, farmers primarily use urea and blended fertilizers but apply the same fertilizer amounts across different crop varieties. There is a notable absence of research on optimal blended fertilizer rates for improved carrot varieties under varying planting years in Northwest Ethiopia (ARARI, 2018). This research aims to address the information gap regarding blended fertilizer application for different carrot varieties, considering the crop’s nutritional and economic importance, with the objective of evaluating the effect of NPSB fertilizer on carrot varieties in a consecutive two-year planting season.

## 2 Materials and methods

### 2.1 Description of the study area

**Figure 1:**
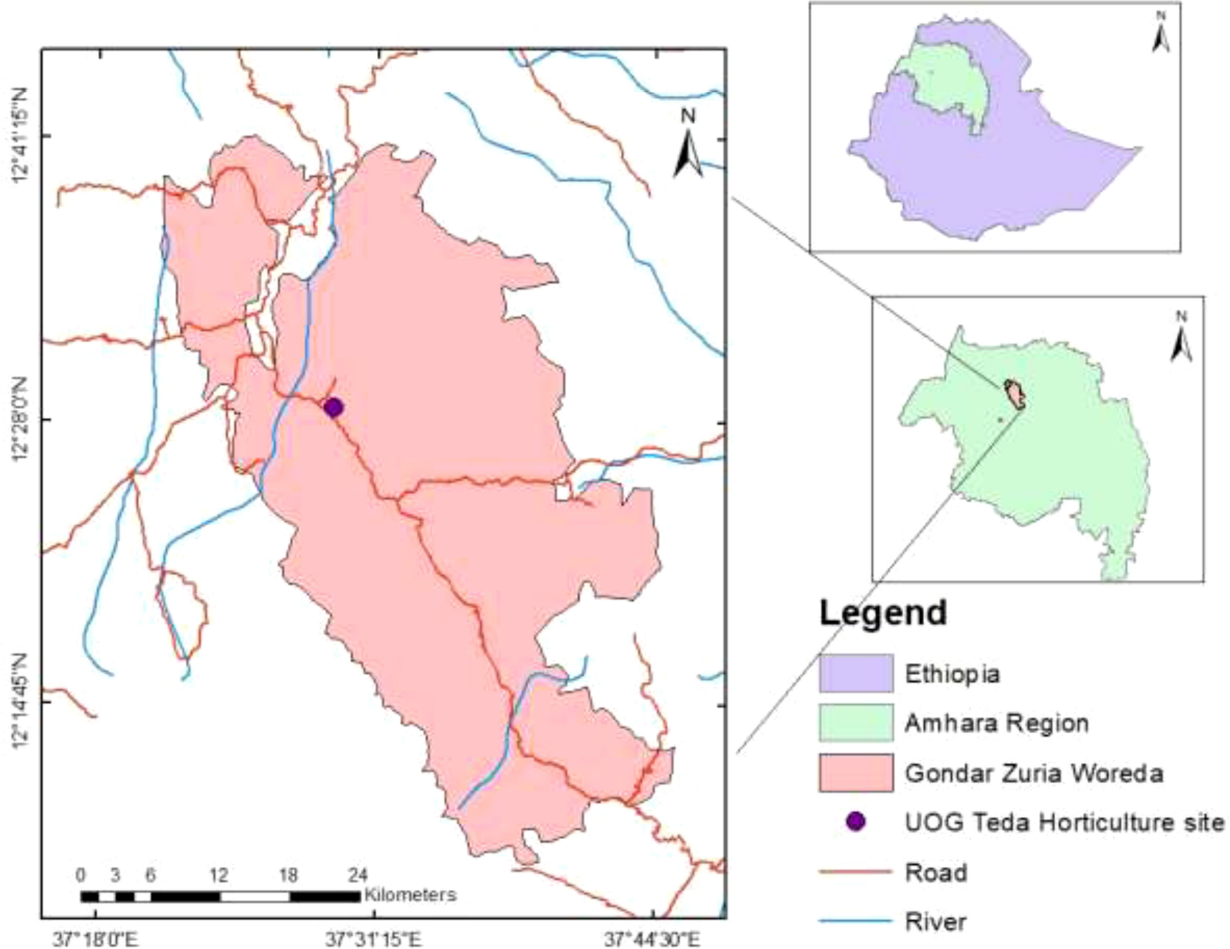
Geographical location of the study area, Gondar, Ethiopia.

The evaluation was conducted in 2021 and 2022 for two consecutive years in the Gondar district under irrigation conditions. The experimental site is located at a latitude of 12°28′N, longitude of 37°29′E, and altitude of 1977 m.a.s. (GPS readings). The average long-term climate data showed that the study area receives a total annual rainfall of 1,058 mm. The study area had a subtropical climate. The minimum and maximum temperatures were 12.7 and 27.3, respectively (Ousman et al., 2018). The climate conditions and precipitation data for 2021 and 2022 in the study area are presented in Appendix Table 5 (**Figure 1**).

### 2.2 Experimental materials and treatments

Two carrot varieties (Nantes and Haramaya-I) and blended fertilizer, NPSB (18.9 N-37.7 P2O5-6.95S-0.1B) and urea (46% N), were used. The varieties were collected from the Holeta Agricultural Research Center for Haramaya-I and the agricultural input delivery shop center for the Nantes varieties. The details of these varieties are presented in **Table 1**.

**Table 1:** Description of varieties.

No.	Varieties	Altitude	Fertilizer rate(kg ha^−1^)	Root color	Root weight (g)
1	Nantes	1,600–2,400	46 P_2_O_5_ and 64 N	Orange	107.2
2	Haramaya–I (AUA-108)	1,600–2,400	46 P_2_O_5_ and 64 N	Deep orange	108

Source: Wassu et al. (2014).

The experiment consisted of a factorial combination of two carrot varieties (Nantes and Haramaya-I) and six NPSB fertilizer levels (0 kg ha^−1^, 40.6 kg ha^−1^, 81.3 kg ha^−1^, 122 kg ha^−1^, 162.3 kg ha^−1^, and 203.4 kg ha^−1^), which were arranged in a randomized complete block design (RCBD) with three replications. The levels of blended fertilizers were set based on the recommended fertilizer for carrots (100 kg DAP and 100 kg urea ha^−1^) (Mohamed et al., 2014). There were a total of 36 experimental plots. Each plot measured 1.8 m2 (1.2 m × 1.5 m), with six rows per plot and 24 plants per row. The distances between the block and plot were 1.5 m and 1 m, respectively. The outer single row on both sides of the plot and one plant at both ends of the rows were considered as border plants. The net plot area was 1.1 m2 (1 m× 1.1 m).

### 2.3 Experimental procedure

The experimental land was oxen plowed three times and disked manually to make the land fine tilth, and the plots were measured and demarked; then, the beds were prepared for each plot. Before sowing, the seeds were mixed with sand (1:1) to increase the volume and avoid dropping too many seeds in one spot. Seeds were sown directly on the prepared land following the rows. Approximately 5 kg of carrot seeds per hectare was used for sowing.

All NPSB rates were applied during sowing. Because nitrogen is a mobile element, urea application was split in two: 50% of the dose during planting, and the remaining 50% was applied 35 days after emergence. Thinning was performed 30 and 40 days after emergence to maintain a spacing of 5 cm between plants (Mehedi et al., 2012; Vithwel and Kanaujia, 2013). Irrigation water was supplied from the beginning to the end by surface irrigation method at 2 days intervals until the seedling established well, and then at 7 days intervals during the experimental period. The plots were irrigated to refill field capacity. Earthing-up was performed every two weeks to cover and protect the roots from sunlight. All other management practices, including weeding and hoeing, were performed as per recommendations.

### 2.4 Data collected

The number of days between sowing and maturity was recorded. Plants showed yellowing of leaves at the time of reaching physiological maturity; carrot roots were harvested (Hailu et al., 2008). Plant height (cm) and 10 randomly selected and tagged plants were measured from ground level to the top of the shoot using a ruler. The number of leaves was counted from 10 randomly selected tagged plants from each experimental plot. Root fresh weight (g) was determined by measuring the fresh weight of 10 randomly collected carrot roots directly after harvesting. Root length (cm), the length of the root of 10 randomly selected and tagged plants, was measured from the base to the marked apex using a ruler, and the mean length of roots was calculated at harvest.

The root diameter (cm) and root diameter of 10 plant samples were measured approximately 2.0 cm below the shoulder using a Vernier caliper, and the average diameter of the root was recorded. Root volume (mm) and average root volume were obtained from 10 randomly selected plants for each treatment by immersing the roots in a beaker containing a known amount of water. The root volume was determined by observing the displacement of water by the root such that the difference was taken as the volume of the root.

Root dry matter (%) was determined by oven-drying the carrot root of 100 g of fresh root weight from each plot and oven-drying at a temperature of 80°C for 72 h or to constant weight, and the weight was measured using a balance.


$$\text{Dry matter}=\frac{dry\;weight\;of\;sample\;}{fresh\;weight\;of\;sample}\times 100$$

Marketable yield (t ha^−1^), roots with no deformities such as cracks, forking, disease, or malformation, and roots without spots and weighing more than 40 g were selected from each net plot area, and the weight was recorded as marketable yield and converted to t ha^−1^ (Appiah et al., 2017). Unmarketable yield (t ha^−1^), roots that showed root cracks, forking, disease, and malformation, and weighed below 40 g were selected from each net plot area, and their weight was recorded as unmarketable yield and converted to t ha^−1^ (Appiah et al., 2017). Total root yield (t ha^−1^) after harvesting, topping, and cleaning of roots, the weight of roots was measured for each net plot. The total yield of the net plot area was converted into t ha^−1^.

### 2.5 Methods of data analysis

Data were subjected to Analysis of Variance (ANOVA) using R-version 4.4.1 software (R core team, 2024).Treatment means were separated by least significant difference (LSD) at the 1 or 5% level of significance, depending on the ANOVA result significance level. Correlation analysis was performed by calculating simple linear correlation coefficients between growth and yield.

## 3 Results and discussion

### 3.1 Levene’s test results for homogeneity of variances among years

Levene’s test for homogeneity of variances between 2021 and 2022 revealed no significant differences in plant height (PH) and days to maturity (DM). However, highly significant differences were observed in number of leaf (NL), fresh weight (FW), Root diameter (RD), and marketable root yield (MRY). Highly significant differences were observed in Root volume (RV) and Dry matter (DM), and significant differences were observed in from Root length (RL), unmarketable yield, and total root yield (TRY) (Appendix Table 1). Consequently, separate mean square ANOVA values were used for plant height and maturity dates in 2021 and 2022 (Appendix Table 2). In contrast, the combined mean square ANOVA values were used for NL, FW, RD, RV, DM, RL, MRY, UMRY, and TRY (Appendix Tables 3, 4).

### 3.2 Effect of planting year on growth, root characteristics, and yield of carrot

The analysis of the results revealed highly significant (P <0.001) influences of both NPSB fertilizer and carrot variety on all parameters except root volume, which was not statistically significant between plant seasons. The result of analysis observed that declines in carrot growth metrics, number of leaves (from 10.31 to 8.53), root length (from 20.09 cm to 16.41 cm), and root diameter (from 2.96 cm to 2.26 cm) between 2021 and 2022 can be attributed to several factors (**Table 2**). Variability in climate is a result of observed droughts in carrots. The compacted root diameter (2.96 cm to 2.26 cm) between 2021 and 2022 can be attributed to several factors. Water stress, soil compaction, and pest pressure are likely to play a significant role in the difference in root growth of carrots.

**Table 2:** Effect of planting year on carrot production.

Year	Number of leaf plant^−1^	Root diameter plant^1^ (cm)	Root length plant^1^ (cm)	Root volume plant^−1^ (mm)	Dry matter (%)	Marketable root yield (t ha^−1^)	Unmarketable yield (t ha^−1^)	Total root yield (t ha^−1^)
2021	10.31^a^	2.96^a^	20.09^a^	89.20^a^	9.53^a^	36.46^a^	7.82^a^	43.30^a^
2022	8.53^b^	2.26^b^	16.41^b^	85.88^a^	7.20^b^	27.98^b^	6.84^b^	36.80^b^
LSD	0.73	0.12	1.31	8.9	0.37	1.23	0.32	1.31
CV	16.4	9.96	15.11	11.40	9.21	8.06	9.17	6.91

Means followed by the same letter(s) in a column and row are not significantly different. *, **, ***, significant at P ≤0.05, P ≤0.01, and P ≤0.001, respectively.

Compacted soils restrict root expansion, potentially explaining the reduced root diameter if soil conditions change owing to increased traffic or cultivation practices (Bengough et al., 2006). Additionally, the decrease in root volume from 89.2 mm in 2021 to 81.82 mm in 2022 could result from soil health issues, water management practices, and genetic variability as well as adverse environmental factors like temperature and light variations. Marketable, unmarketable, and total root yields were significantly different between the two planting seasons.

Higher marketable and total yields of carrot (36.46 t ha^−1^ and 43.30 t ha^−1^) were recorded from the planting year of 2021, respectively. Lower marketable and total yield (27.98 t ha^−1^ and 36.80 t ha^−1^) were revealed from the planting year of 2022, respectively. This indicated that carrot production performance was influenced by the production season (Tolessa, 2022).

### 3.3 Effect of planting year and NPSB fertilizer rates on plant height and days to maturity of carrot varieties

The analysis of the results highlights the significant influence of both NPSB fertilizer and carrot variety on days to maturity. NPSB fertilizer exhibited a highly significant effect (P <0.001), while the variety showed a significant effect (P <0.05). However, the interaction between these factors was not significant (P >0.05) across either planting year.

In 2022, the control group (no fertilizer) matured the earliest at 91.55 days, contrasting with the delayed maturity observed at higher NPSB rates, particularly 115.61 days at 203.4 kg ha^−1^ (**Table 3**). Similarly, in 2021, the control group matured in 93.95 days, with increasing fertilizer rates extending maturity by 24.04 days in 2022 and 18.72 days in 2021, likely due to nitrogen’s role in prolonging vegetative growth. Varietal differences were also evident, with ‘Haramaya-I’ maturing earlier than ‘Nantes,’ requiring 101.55 days and 102.82 days in 2021 and 2022, respectively. In contrast, ‘Nantes’ took longer, maturing at 104.56 days and 105.30 days for the same years. These variations are attributed to genetic differences and responses to environmental conditions, findings by Melese et al. (2018).

**Table 3:** Main effect means of blended NPSB fertilizer rates and varieties on days to maturity and plant height in two consecutive years.

Treatments	Maturity date plant^−1^ (2021)	Maturity date plant^−1^ (2022)	Plant height plant^−1^ (2021)	Plant height plant^−1^(2022)
NPSB kg ha^−1^				
0	93.95^d^	91.55^d^	28.90^d^	29.57^c^
40.6	97.74^cd^	97.66^c^	33.66^c^	32.00^b^
81.3	100.47^bc^	101.91^b^	35.40^c^	32.90^b^
122	103.85^b^	105.28^b^	37.03^bc^	33.70^ab^
162.3	110.22^a^	112.32^a^	39.97^ab^	35.00^a^
203.4	112.67^a^	115.61^a^	41.17^a^	35.63^a^
LSD	4.02	3.80	3.37	2.05
Varieties				
Haramaya-1	101.55^b^	102.82^a^	37.45^a^	33.96a
Nantes	104.56^a^	105.30^a^	34.59^b^	32.31b
LSD	2.32	2.19	1.95	1.19
CV (%)	3.26	3.05	7.81	5.18

Means followed by the same letter(s) in a column and row are not significantly different. *, **, ***, significant at P ≤0.05, P ≤0.01, and P ≤0.001, respectively.

Plant height was significantly affected by both NPSB fertilizer application (P <0.001) and variety (P <0.05), but no interaction effect was observed (P >0.05). The tallest plants were recorded at the highest fertilizer rate of 203.4 kg ha^−1^ in both years, with heights of 41.47 cm in 2021 and 35.63 cm in 2022, compared with shorter heights in the control group. These increases in height are likely due to enhanced photosynthesis from nitrogen and phosphorus, which are crucial for growth during periods of high metabolic activity, as well as the roles of sulfur in chlorophyll formation (Hatwar et al., 2013). This aligns with previous research indicating that plant height was not significantly affected by the interaction between carrot variety and fertilization rates (Nikmatullah et al., 2021).

### 3.4 Effect of NPSB fertilizer on number of leaves of carrot varieties

The combined analysis of variance indicated that the number of leaves in plant^−1^ was significantly (P <0.001) affected by the main effect of NPSB fertilizer and variety. However, the interaction between these factors was not significant (P >0.05). The largest number of leaves per plant (10.51) was recorded in 203.4 NPSB kg ha^−1^, whereas the lowest number of leaves per plant (8.57) was recorded in the unfertilized plot (**Table 4**). The reduced leaf number at a lower level of fertilizer might be due to the lack of sufficient nutrients and assimilation for growth, whereas the increase in leaf number per plant at a higher rate of NPSB fertilizer could be attributed to the availability of macro- and micronutrients that permit vigorous growth of leaves. The current result is related to the findings of Hailu et al. (2008), who reported that the application of orga and urea had a significant effect on the number of leaves. Pongener et al. (2018) also showed that the maximum number of leaves (8) was obtained with 1.5% Zn + 2% B + 2% Cu t ha-1 and the minimum (6.5) was obtained from the control. A larger number of leaves per plant (9.53) was recorded in Haramaya-I, whereas a lower number of leaves per plant (9.26) was recorded in the Nantes variety, which was not a significant difference between varieties.

**Table 4:** Effect of NPSB on number of leaves of carrot variety.

Treatments	Number of leaves per plant^−1^
NPSB kg ha^−1^	
0	8.57^c^
40.6	8.84^bc^
81.3	9.04^bc^
122	9.67^abc^
162.3	9.87^ab^
203.4	10.51^a^
LSD	1.27
Varieties	
Haramaya-1	9.53^a^
Nantes	9.26^a^
LSD	0.73
CV (%)	16.4

Means followed by the same letter (s) in columns and rows are not significantly different according to the LSD at 5% levels of significance.

This result agrees with the findings of Biratu et al. (2020), who observed that the number of leaf plants was significantly influenced by variety.

### 3.5 Root characteristics

#### 3.5.1 Root fresh weight

The combined analysis of variance revealed that the root weight of carrots was significantly (P <0.001) affected by different rates of NPSB fertilizer and variety, and their interaction showed a significant effect (P <0.05). The highest average root weight (134.48 g plant^−1^) was obtained from Haramaya-I variety and application of 162.3 NPSB kg ha^−1^, while the lowest average root weight (57.13 g plant^−1^) was recorded by the Nantes variety from the unfertilized plots, which was at par with the value (61.55 g plant^−1^) recorded by the Haramaya-I variety when it was unfertilized (**Table 5**).

**Table 5:** Interaction effect of NPSB on root fresh weight of carrot varieties.

Treatments	Root fresh weight (g plant^−1^)	
NPSB rate Kg ha^−1^	Haramaya-1	Nantes
0	61.55^h^	57.13^i^
40.6	81.35^g^	83.41^g^
81.3	93.72^f^	91.30^f^
122	132.24^ab^	112.11^e^
162.3	134.48^a^	127.35^cd^
203.4	130.00^bc^	125.04^d^
LSD	3.31	
CV (%)	2.77	

Means followed by the same letter (s) in columns and rows are not significantly different according to the LSD at 5% levels of significance.

The root weight of carrot had increased as blended fertilizer increased from 0 kg ha^−1^ to 162.3 kg ha^−1^ NPSB fertilizer. This could be due to increased vegetative growth and, hence, increased food production and assimilation into parts. However, with the excess application of nitrogen fertilizer, more aboveground vegetative growth is favored over root growth, resulting in low root weight. In line with this, Hailu et al. (2008) justified that the general trend of increasing and then decreasing carrot root weight was evident due to the increased rate of nitrogen fertilizer application.

The higher root weight per plant was due to the larger number of leaves for photosynthesis and efficient utilization of photosynthesis, which might have enhanced the weight of the root (Patel et al., 2015). This result was consistent with that reported by Ladumor et al. (2020). Variations in root weight among different carrot varieties might be due to varietal differences.

#### 3.5.2 Root length

The combined analysis of the main effects of NPSB fertilizer rate and variety showed a highly significant (P <0.001) effect on the root length of carrots, and their interaction showed no significant effect (P <0.05).

The root length was increased as NPSB increased from 0 kg NPSB kg ha^−1^ to 162.3 kg NPSB kg ha^−1^ and fluctuated beyond 162.3 kg NPSB kg ha^−1^ in both varieties. In line with this, Kreuzmann et al. (2007) reported that the highest root length (17.19 cm) per plant was found with 100 kg ha^−1^ of nitrogen and the lowest root length (15.39 cm) was recorded in the untreated fertilizer.

The current experiment is also in agreement with Elwan et al. (2018) on the application of ammonium sulfate and spraying with a mixture of micronutrients that had the highest root length among the carrot varieties.

#### 3.5.3 Root diameter

The analysis of variance indicated that NPSB fertilizer rates and varieties had highly significant (P <0.001) and highly significant (P <0.01) effects on root diameter, whereas the interaction of both factors did not significantly affect root diameter.

Significantly, the highest root diameter per plant (3.38 cm) was recorded from 162.3 kg NPSB kg ha^−1^, followed by 3.27 cm obtained from 203.4 kg NPSB kg ha^−1^, which are statistically similar, while the smallest root diameter of 1.78 cm was recorded from the control (**Table 6**). The highest root diameter per plant at higher rates of NPSB blended fertilizer might be due to the phosphorus fertilizer stimulating root diameter growth. The critical role of phosphorus in carrot plants is root diameter growth and root development (Roy et al., 2006). Carrots require adequate available phosphorus for normal growth. This result is in line with the findings of Pongener et al. (2018), who reported that the maximum root diameter recorded was 30.50 mm through the combined application of macro- and micronutrients, whereas the minimum was obtained through the control with 24.71 mm. Sultana et al. (2015) confirmed that a higher percentage application of micronutrient combination has the value of increasing the root diameter of carrot than without nutrients applied.

**Table 6:** Effect of NPSB on root diameter, root length, and root volume of carrot variety.

Treatments	Root diameter plant^−1^ (cm)	Root length plant^−1^ (cm)	Root volume plant^−1^ (mm)
NPSB kg ha^−1^			
0	1.78^d^	13.09^d^	50.50^e^
40.6	1.97^d^	17.27^c^	76.50^d^
81.3	2.27^c^	18.15^bc^	87.45^cd^
122	3.17^b^	19.58^ab^	92.84b^c^
162.3	3.38^a^	20.93^a^	110.60^a^
203.4	3.27^ab^	20.46^a^	107.33^ab^
LSD	0.21	2.27	15.41
Varieties			
Haramaya-1	2.75^a^	19.20^a^	93.26^a^
Nantes	2.48^b^	17.29^b^	81.82^b^
LSD	0.12	1.31	8.9
CV (%)	9.96	15.11	11.40

Means followed by the same letter (s) in columns and rows are not significantly different according to the LSD at 5% levels of significance.

Regarding carrot varieties, a significantly higher root diameter (2.75 cm) was recorded from Haramaya-1, whereas a lower root diameter (2.48 cm) was obtained from the Nantes variety. This might be attributed to the inherent characteristics of the cultivars and environmental conditions. This result was similar to that of Biratu et al. (2020), who reported that the highest root diameter (3.05 cm) was recorded in the Haramaya-I variety.

#### 3.5.4 Root volume

The analysis of variance revealed that root volume was highly significant (P <0.001), influenced by different levels of NPSB fertilizer, and highly (p <0.01) affected by variety, but their interaction did not.

The maximum root volume (110.60 ml) was obtained from carrot plants, which received 162.3 kg NPSB kg ha^−1^, whereas the minimum root volume (50.50 ml) was obtained from the control. Excess application of inorganic nitrogen fertilizer did not improve root volume. Conversely, a decrease in root volume was observed because of excess nitrogen fertilizer application above the recommended rate. Melese (2016) found a significant difference in the root volume of carrots due to the application of different rates of nitrogen and orga.

In contrast, the carrot varieties showed a significant difference in root volume. The highest root volume (93.26 mm) was recorded in the Haramaya-I variety, whereas a lower root volume (81.82 mm) was obtained in the Nantes variety.

#### 3.5.5 Dry matter

The interaction effects of NPSB fertilizer and varieties on the dry matter of roots were highly significant (P <0.001). The maximum value of 13.67% was obtained at 162.3 kg ha^−1^ from the Haramaya-I variety and the minimum value of 5.17% was obtained in the control from the Nantes variety (**Table 7**). The root dry matter increased from 5.17% to 13.67% as NPSB fertilizer increased from 0 NPSB kg ha^−1^ to 162.3 NPSB kg ha^−^ *^1^* with the Haramaya-1 variety, while in the Nantes variety, the dry matter increased from 4.75% to 10.20% as NPSB fertilizer increased from 0 NPSB kg ha^−1^ to 203.4 NPSB kg ha^−^ *^1^*.

**Table 7:** Interaction effect means of root dry matter (RDM) of carrot as affected by blended NPSB fertilizer rates and varieties.

Treatments	Root dry matter (%)	
NPSB rate Kg ha^−1^	Haramaya-1	Nantes
0	5.168^i^	4.748^i^
40.6	6.701^gh^	6.101^h^
81.3	8.335^ef^	7.068^g^
122	9.228^de^	7.56^fg^
162.3	13.67^a^	9.768^cd^
203.4	11.87^b^	10.20^c^
LSD	0.89	
CV (%)	9.21	

Means followed by the same letter (s) in columns and rows are not significantly different according to the LSD at 5% levels of significance.

This might be due to the carrot plants’ uptake of more nutrients and accumulation of higher dry matter in the storage organ. The current results agree with the findings of Kreuzmann et al. (2007) that variety and nitrogen sources affect the dry matter content. Kumar et al. (2014) and Grubinger (1999) also reported that nitrogen and phosphorus fertilizers increased dry matter production. El-Sayed et al. (2011) found that phosphorus rate had a significant effect on the storage of root dry matter. Along with nitrogen and phosphorus, boron significantly increases the dry matter production of carrots (Solanki et al., 2018).

### 3.6 Yield parameters

The interaction effect of varieties with NPSB fertilizer rates resulted in highly significant (p <0.001) changes in the mean of the marketable, unmarketable, and total yield of carrots.

The Haramaya-I variety yielded the highest marketable (50.77 t ha^−1^) and total root yield (55.32 t ha^−1^) when treated with 162.3 kg NPSB ha^−1^) fertilizer (**Table 8**).

**Table 8:** Interaction effect means of marketable unmarketable and total root yield of carrot as affected by NPSB blended fertilizer rates and varieties.

Treatments	Marketable root yield (t ha^−1^)		Unmarketable yield (t ha^−1^)		Total root yield (t ha^−1^)		
NPSB Kg ha^−1^	Haramaya1	Nantes	Haramaya1	Nantes	Haramaya1		Nantes
0	20.65^g^	19.52^g^	9.37^b^	10.77^a^	30.79^g^		30.52^g^
40.6	26.52^ef^	23.75^f^	8.57^cd^	8.87^bc^	35.59^ef^		32.79^fg^
81.3	31.25^d^	27.35^e^	7.90^de^	8.54^cd^	38.49^de^		36.72^e^
122	42.55^b^	34.15^cd^	5.94^g^	7.50^ef^	48.99^b^		39.960^cd^
162.3	50.77^a^	34.85^c^	4.04^h^	5.34^g^	55.32^a^		42.56^c^
203.4	43.32^b^	31.95^cd^	4.37^h^	6.74^f^	48.19^b^		40.69^cd^
LSD	3.02		0.77		3.22		
CV (%)	8.06		9.17		6.91		

Means followed by the same letter(s) in a column are not significantly different according to the LSD at 5% level of significance.

In contrast, untreated Nantes and Haramaya-I varieties produced the lowest yields (20.65 t ha^−^¹ and 30.79 t ha^−^¹, respectively). This suggests that increasing NPSB fertilizer enhances total root yield, likely because of the beneficial effects of nitrogen, phosphorus, sulfur, and boron on carrot growth.

Phosphorus is crucial for root development and energy transfer (Roy et al., 2006), which is consistent with the findings of Mubashir et al. (2010) and Ahmed (2014), who emphasized the significance of nitrogen and phosphorus on marketable yields. Additionally, sulfur improves nutrient absorption and photosynthesis, thereby contributing to higher yields (Zhou et al., 2005).

Micronutrient applications further enhance marketable yields (Tawfic et al., 2014). These results confirm that carrot yield is influenced by variety and fertilizer application rates (Nikmatullah et al., 2021).

The Haramaya-I variety outperformed Nantes, potentially due to genetic differences, climate, and cultural practices, supporting Wassu et al. (2014), who found Haramaya-I superior in root production. On the other hand, significantly the highest unmarketable root yield (10.77 t ha^−1^) was recorded from the control with the Nantes variety, while the smallest value (4.37 t ha^−1^) was recorded from the Haramaya I variety received with 203.4 kg NPSB ha^−1^. The increase in unmarketable yield may be linked to inadequate nitrogen, which is crucial for compounds such as chlorophyll and enzymes essential for plant growth (Brady and Weil, 2002). Additionally, low phosphorus levels can lead to poor root development, further reducing the carrot yield (Kreutzmann et al., 2007). Boron deficiency also plays a significant role by restricting stomatal opening and increasing solute leakage, which diminishes the photosynthetic potential and contributes to higher unmarketable yields (Subba et al., 2017).

Moreover, excess nitrogen fertilizer can lead to increased branching and cracking severity in carrots, resulting in higher unmarketable yields (Hartz et al., 2005). Mehedi et al. (2012) noted significant variations in cracked root percentages due to different nitrogen levels. Among the varieties, the Haramaya-I variety exhibited the lowest unmarketable yield, whereas the Nantes variety had the highest. This disparity may stem from a correlation between growth traits and unmarketable yield; varieties with more leaves and greater height tend to have lower unmarketable yields, as leaf number is strongly negatively correlated with unmarketable yield (Ahmed et al., 2014).

## 4 Correlation analysis

**Figure 2:**
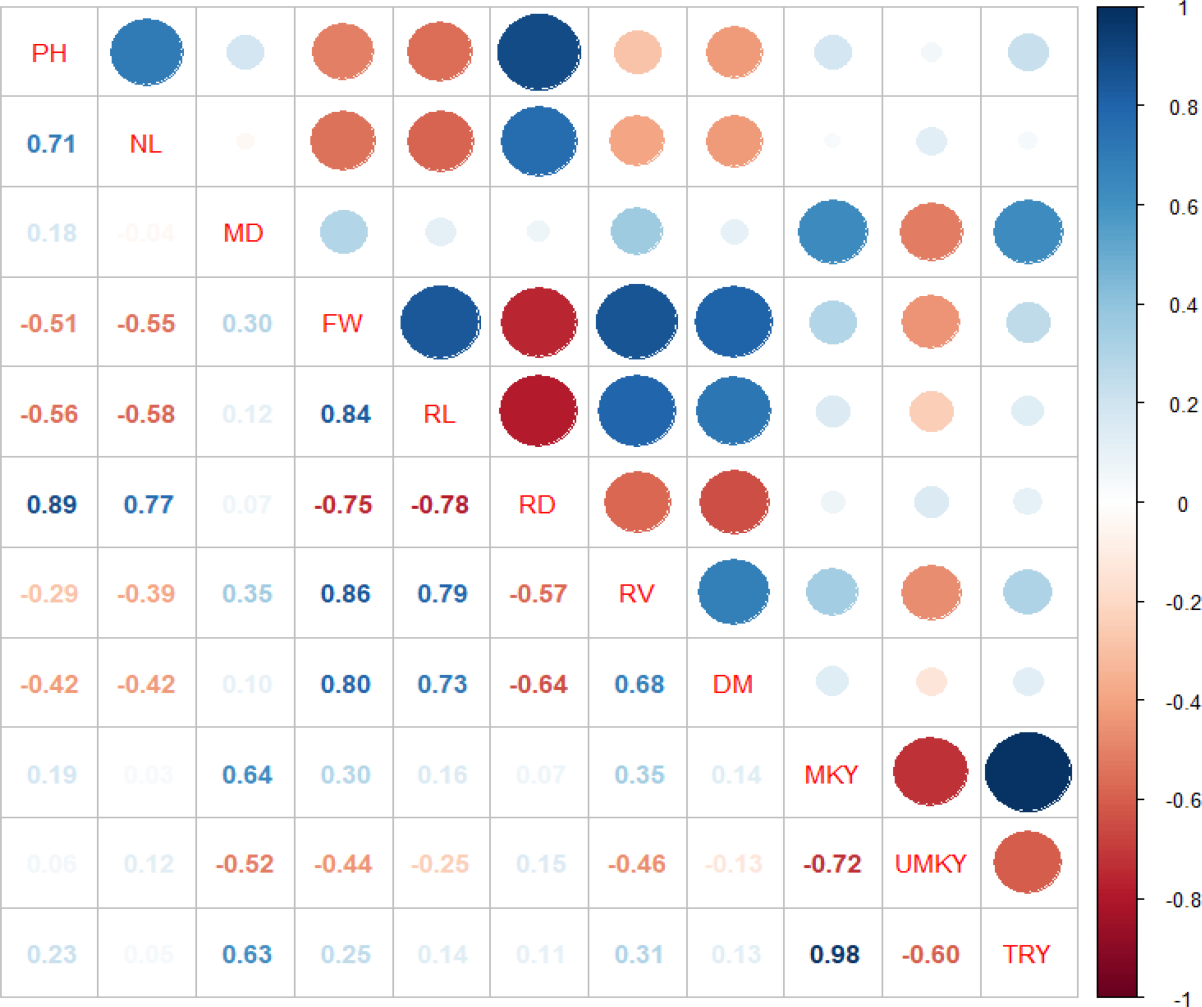
Simple correlations (r) among growth, root characteristics, and yield of carrot under different rates of NPSB fertilizer rate and varieties. PH, plant height; NL, number of leaf; MD, maturity date; FW, root fresh weight; RL, root length; RD, root diameter; RV, root volume; DM, dry matter; MRY, marketable root yield; UMRY, unmarketable root yield; TRY, total root yield.

The correlation coefficient (r) values computed to determine the association between the growth parameters and root yield are shown in **Figure 2**. The correlation values explained the apparent association between the growth parameters and root yield, indicating the magnitude and direction of the association and relationships.

Days to maturity and root volume had a strong positive correlation with marketable and total root yields (p <0.001), implying that an increase in root volume enhanced total root yield and marketable root yield (**Figure 2**).

Root fresh weight also showed a significant positive correlation with marketable (r = 0.3***) and total (r = 0.25***) root yield. However, it had a strong negative association with unmarketable root yield (r = −0.44***).

## 5 Conclusion

The results of this study showed an increase in yield when 162.3 NPSB kg ha^−1^ of fertilizer was combined with both varieties to obtain the maximum yield. A general trend of increased root yield of carrots due to the increased rate of NPSB fertilizer application was observed. Nevertheless, this was apparent only up to the rate of 162.3 NPSB kg ha^−1^. There was a significant difference between the two varieties. Producing the Haramya-I variety was better because it had a higher marketable and total yield than that of the Nantes variety. Year one (2021) was the best season for carrot production compared with year two (2022). As a result, carrot production performance is influenced by the production season and the inherent characteristics of the genotype. Therefore, it is essential to consider these aspects in carrot production regions.

## Data Availability

The original contributions presented in the study are included in the article/supplementary material. Further inquiries can be directed to the corresponding author.

## Appendix

### Appendix

**Appendix Table 1:** Levene’s Test for Homogeneity of Variance between 2021 and 2022 planting year as affected by NPSB fertilizer on carrot variety.

Grouping variables	F values for Levene’s test										
	PH	NL	MD	FW	RL	RD	RV	DM	MKY	UMKY	TRY
Year	1	0.00032***	0.14^ns^	0.0023***	0.015*	0.0002***	0.0023**	0.0038**	0.001***	0.021*	0.038*

PH, plant height; NL, number of leaf; MD, maturity date; FW, root fresh weight; RL, root length; RD, root diameter; RV, root volume; DM, dry matter; MRY, marketable root yield; UMRY, unmarketable root yield; TRY, total root yield.

**Appendix Table 2:** Separate mean square values of plant height and maturity date as influenced by the main and interaction effects of varieties and NPSB fertilizer rates for the experiment conducted in 2021 and 2022.

Source of variation	DF	Mean squares for 2021 year		Mean squares for 2022 year	
		Plant height	Maturity date	Plant height	Maturity date
Replication	2	25.534^ns^	7.352^ns^	13.12^*^	25.24^ns^
Variety	1	73.96^**^	92.480^**^	24.33^**^	55.50^*^
NPSB	5	119.65^***^	314.88^***^	28.938^***^	486.23***
Variety*NPSB	5	21.363^ns^	24.623^ns^	9.233^ns^	24.81^ns^
Error	22	7.922	11.292	2.94	10.08
CV (%)		7.81	3.26	5.18	3.05

**Appendix Table 3:** Combining analysis of over year mean squares of number of leaf and root characteristics.

Source of variation	DF	Number of leaf	Root fresh weight	Root diameter	Root length	Root volume	Root dry matter
Year	1	57.031***	13454.6***	8.82***	243.51***	198.6^ns^	97.720***
Variety	1	1.742ns	684.4***	1.34***	65.380**	2355.8*	45.252***
NPSB	5	6.356*	10085.6***	6.25***	99.63***	5868.6***	82.260***
Year*rep	4	36.28***	0.3^ns^	0.044^ns^	1.52^ns^	555.5^ns^	0.006
Year*variety	1	0.00	0.00	0.00	0.001	0.00	0.00
Year NPSB	5	0.00	0.00	0.00	0.001	0.00	0.00
Variety*NPSB	5	1.033ns	134.5***	0.116^ns^	12.918^ns^	1610.8^ns^	4.680***
Year *variety*NPSB	5	0.00	0.00	0.00	0.001	0.00	0.00
Error	44	2.399	8.1	0.0679	7.603	351.1	0.594
CV (%)		16.4	2.77	9.96	15.11	11.41	9.21

DF, degree of freedom; ns, non-significant, *, **, ***, significant at P ≤ 0.05, P ≤ 0.01and P ≤ 0.001, respectively.

**Appendix Table 4:** Combining analysis of over year mean square of yield parameters.

Source of variation	DF	Marketable yield	Unmarketable yield	Total root yield
Year	1	1294.39^***^	17.287^***^	760.50^***^
Variety	1	1215.24^***^	28.627^***^	870.84^***^
NPSB	5	870.98^***^	49.528^***^	519.46^***^
Year*rep	4	0.67^ns^	0.120^ns^	0.64^ns^
Year*variety	1	0.00	0.00	00.00
Year NPSB	5	0.00	0.00	00.00
Variety*NPSB	5	97.16^***^	1.859^**^	84.89^***^
Year *variety*NPSB	5	0.00	0.00	0.00
Error	44	6.75	0.445	7.65
CV (%)		8.06	9.17	6.91

DF, degree of freedom; ns, non-significant, *, **, ***, significant at P ≤ 0.05, P ≤ 0.01and P ≤ 0.001, respectively.

**Appendix Table 5:** The climate conditions and precipitation data for the years 2021 and 2022 in the study area.

PARAMETER	YEAR	JAN	FEB	MAR	APR	MAY	JUN	JUL	AUG	SEP	OCT	NOV	DEC	ANNUAL
Relative humidity (%)	2021	42.19	43.5	34.56	47.62	65.06	67.19	89.81	86.88	79.25	74.69	64.81	53.31	62.56
	2022	58.88	47.69	37.69	47.75	52.25	75.38	89.31	88.31	80.94	74.75	62.56	54.62	64.31
Temp. maximum (°C)	2021	28.59	29.42	32.56	32.09	29.55	29.72	23.69	23.87	24.73	23.93	26.34	28.26	32.56
	2022	27.03	30.1	32.69	32.43	30.82	30.37	22.56	23.61	24.22	24.74	26.09	26.39	32.69
Temp. minimum (°C)	2021	10.73	10.26	13.54	15.01	14.68	14.54	12.95	13.37	13.37	11.16	11.2	8.9	8.9
	2022	10.58	12.09	15.58	14.58	15.86	14.19	12.71	13.08	13.6	10.89	10.48	9.99	9.99
Precipitation (mm/day)	2021	0	0.38	0	5.07	6.53	11.31	37.24	20.55	16.07	2.14	0.44	0.76	8.44
	2022	1.14	0.11	0.37	6.45	2.16	19.73	32.2	12.42	9.97	5.39	0.13	0.22	7.57
Light (PAR total) (w/m^2^)	2021	115.13	116.66	131.67	117.84	116.3	114.4	83.56	99.09	108.73	110.11	108.67	108.89	110.86
	2022	107.53	116.48	130.69	128.51	123.6	108.02	85.59	86.09	102.94	109.16	112.73	104.33	109.55

## References

[B1] Ahmed A. Sambo B. E. Arunah U. L. Odion E. C. (2014). Response of farmyard manure and inorganic fertilizers for sustainable growth of carrot (*Daucus carota* L.) in Northern Nigeria. J. Agric. Vet. Sci. 7, 26–33. doi: 10.9790/2380-07221825

[B2] Alemayehu M. Tessafa F. Bizuayehu S. Ayele B. (2015). Amhara region horticulture development strategy (ARHDS) (2015-2019) (Bahir Dar, Ethiopia: College of Agriculture and Environmental Sciences Bahir Dar University).

[B3] Appiah F. K. Addo S. J. Opoku A. (2017). Growth and yield response of carrot (*Daucus carota* L) to different green manures and plant spacing. J. Biol. Agric. Healthc. 7, 16–23.

[B4] ARARI (Amhara Regional Agricultural Research Institute) (2018). Participatory Agricultural Production Systems Analysis of AGP-II Districts in North Gondar Zone: Implications for Research and Development (Amhara Regional office of agriculture Ethiopia: Gondar Agricultural Research Center).

[B5] ATA (Agricultural Transformation Agency) (2016). EthioSIS.Soil fertility and fertilizer recommendation atlas (Adiss Ababa, Ethiopia: Ministry of Agriculture (MoA)).

[B6] Bengough A. G. Mullins C. E. Waisel Y. (2006). The effect of soil compaction on root diameter of carrot (Daucus carota L.). Plant Soil 280, 1–10. doi: 10.1007/s11104-005-9001-2

[B7] Biratu W. Molla B. Abebe H. Gebremeskel H. (2020). Evaluation of Carrot (*Daucus carrota*) Cultivarss for Growth, Root Yield and Yield Related Traits at Adigrat and Atsbi, Eastern Zone of Tigray Region. J. Natural Sci. Res. 10, 14–18.

[B8] Brady Weil (2002). The nature and properties of soils. Thirteenth edition (Delhi, India: Pearson Education Asia).

[B9] CGAO (Central Gondar zone agriculture office). (2021). The Amhara region, central Gondar zone agriculture office annual crop data. (Accessed December 05, 2021).

[B10] CSA (Central Statistical Agency) (2020). The federal democratic republic of Ethiopia central statistical agency agricultural sample survey 2019/20 (2012 E.C.) volume I, report on area and production of major crops, Statistical Bulletin 587 (Addis Ababa, Ethiopia).

[B11] De Lannoy G. (2001). Carrot Crop Production in Tropical Africa. Ed. Raemaekers R. H. (Brussels, Belgium: Directorate-General for International Corporation), 480–485.

[B12] El-Sayed H. E. A. Saif-el-Dean A. Ezzat S. El-Morsy A. H. A. (2011). Responses of productivity and quality of Sweet potato to phosphorus fertilizer rates and application methods of the humic acid. Int. Res. J. Agric. Sci. Soil Sci. 1, 383–393.

[B13] Elwan M. K. Hamed E. A. Youssef M. K. Soliman S. M. (2018). Plant growth, root yield and nutrients content of carrot as affected by the source of nitrogen and application of micronutrients. J. Suez Canal Univ. 7, 15–24.

[B14] FAO (Food and Agriculture Organization of the United Nations) (2019). FAOSTAT databases. Available online at: http://www.fao.org/faostat/en/#data/QC (Accessed April 6, 2021).

[B15] Grubinger V. P. (1999). Sustainable Vegetable Production from start-up to market (Ithaca, New York: Soil Fertility Management. Natural Resource, Agriculture and Engineering Service).

[B16] Hailu S. Seyoum T. Dechassa N. (2008). Effect of combined application of organic P and inorganic N fertilizers on post-harvest quality of carrot. African J. Biotechnol. 7, 2187–2196.

[B17] Hart J. M. Butler M. D. (2003). Seed carrot aboveground biomass and nutrient accumulation. (Mardas, Oregon: 2001/2002 growing seed)

[B18] Hartz T. K. Johnstone P. R. Nunez J. J. (2005). Production environment and nitrogen fertility affect carrot cracking. Hortic. Sci. 40, 611–615.

[B19] Hatwar G. P. Gondane S. M. Urkade S. M. Gahukar O. V. (2013). Effect of micronutrients on growth and yield of chilli. Soils Crops 13, 123–125.

[B20] Heinonen M. (1990). Carotenoids and Provitamin A. activity of carrot (*Daucus carota* L.) cultivars. J. Agric. Food Chem. 38, 609–612.

[B21] Kreutzmann S. Thybo A. K. Bredie W. L. (2007). Training of a sensory panel and profiling of winter hardy and colored carrot genotypes. Food Qual. Prefer. 18, 482–489. doi: 10.1016/j.foodqual.2006.05.009

[B22] Kumar P. Meghwal P. R. Painuli D. K. (2014). Effect of organic and inorganic nutrient sources on soil health and quality of carrot. Indian J. Hortic. 71, 222–226.

[B23] Ladumor B. M. Nandre M. K. Sharma V. R. Wankhade Joshi (2020). Performance of Different Varieties of Carrot (*Daucus carota* L.) with Respect Yield, Quality and Chemical Compositions under varying Sowing Times. Int. J. Curr. Microbiol. Appl. Sci. 9, 126–132. doi: 10.20546/ijcmas.2020.902.015

[B24] Mehedi T. A. Siddique M. A. Shahid S. B. (2012). Effects of urea and cow dung on growth and yield of carrot. J. Bangladesh Agric. Univ. 10, 9–13.

[B25] Melese K. Teklay K. Shelema A. Gebra Michael T. Kidane H. Gebra Kiros B. (2018). On-farm demonstration of improved carrot (*Daucus carota* L.) variety in Emba Alaje District, Northern Ethiopia. Int. J. Agric. Biosci. 7, 218–221.

[B26] Melesse B. (2016). Effects of Combined application of organic P and inorganic N fertilizers on yield of Carrot (*Daucus Carota* L.). J. Agric. Res. Technol. 2, 35–39. doi: 10.19080/ARTOAJ.2016.01.555581

[B27] Mubashir M. Malik S. A. Khan A. A. Ansari T. M. Wright S. Brown M. V.. (2010). Growth, yield and nitrate accumulation of irrigated carrot and okra in response to nitrogen fertilization. J. Biotechnol. 42, 2513–2521.

[B28] Nikmatullah A. Zawani K. Muslim K. Sarjan M. (2021). Responses of four varieties of carrot plant (*Daucus carota* L.) grown in medium latitude to different dosages of fertilization. The7^th^ International Conference on Sustainable Agriculture and Environment. IOP Conference Series: Earth and Environmental Science. (Indonesia: West Nusa Tenggara). 637:1–6. doi: 10.1088/1755-1315/637/1/012079

[B29] Ousman Y. Alemayehu G. Ayalew D. (2018). Effects of supplemental irrigation on yield and yield attributes of chickpea (*Cicerarietinum* L.) in Western Ethiopia. Ethiopian J. Sci. Technol. 11, 97–112. doi: 10.4314/ejst.v11i2.2

[B30] Patel H. T. Sharma M. K. Varma L. R. (2015). Effect of planting date and spacing on growth, yield and quality of beet root (Beta vulgaris L.) cultivars under north Gujarat climatic conditions. J. Agric. Sci. Resour. 5, 119–126.

[B31] Pongener I. Daniel S. Oshin. S. Marak C. Suren A. Srinivas K.. (2018). Effect of different micro-nutrients (B, Zn & Cu) on the growth, yield and tuber quality of Carrot (*Daucus carota* L.) under Teak-based Agroforestry system. J. Pharmacogn. Photochem. 7, 2326–2328.

[B32] R core team (2024). R: A language and environment for statistical computing. (Vienna, Austria: R foundation for Statistical computing). Available at: https://www.R-project.org/ (Accessed June 10, 2024).

[B33] Roy R. N. Finck A. Blair G. J. Tandon H. L. S. (2006). Plant Nutrition for Food Security, A guide for integrated nutrient management, FAO fertilizer and nutrition Bulletin 16 (Rome, Italy: Food and Agriculture Organization of the United Nations FAO).

[B34] Sharma K. D. Karki S. Thakur N. S. Attri S. (2012). Chemical composition, functional properties and processing of carrot-a review. J. Food Sci. Technol. 49, 22–32. doi: 10.1007/s13197-011-0310-7 23572822 PMC3550877

[B35] Solanki S. Singh L. Singh Y. (2018). Differential response of vegetable crops to boron application. Ann. Plant Soil Res. 20, 239–242.

[B36] Subba K. S. Chattopadhyay S. B. MondaL R. Dukpa. P. (2017). Carrot root production as influenced by potassium and boron. Crop Resour. 52, 41–44.

[B37] Sultana S. Muhmood A. Shah S. H. S. S. Saleem N. ,. A. Ahmed A. Z. Wakee A. (2015). Boron uptake, yield and quality of carrot (*Daucus carota* L.) in response to boron application. Int. J. Plant Soil Sci. 8, 1–5. doi: 10.9734/IJPSS/2015/19667

[B38] Tabor G. Yesuf M. (2012). Mapping the Current Knowledge of Carrot Cultivation in Ethiopia. (Conference, Denmark). 1–20.

[B39] Tawfic S. F. Aziz A. R. Eanar A. E. (2014). Effect of planting date and sulfur fertilizer on yield and quality of sugar beet under newly reclaimed soils. J. Agric. 5, 1547–11556.

[B40] Tolessa D. Tadesse D. ,. B. Abewoy D. Gudisa H. ,. M. (2022). Evaluation of Carrot (Daucus carota Var.Sativa) Varieties for Yield and Related Traits under Wondo Genet and Negelle Arsi Conditions. World J. Agric. Sci. 18, 27–315. doi: 10.5829/idosi.wjas.2022.27.31

[B41] Vithwel S. Kanaujia P. (2013). Integrated nutrient management on productivity of carrot and fertility of soil. J. Agric. 11, 173–181.

[B42] Wassu M. Tewodros B. Nigussa D. Kebede W. Mulatua H. Bekele A. (2014). Registration of “Haramaya I” Carrot (*Daucus carota* L.) Variety. East Afr. J. Sci. 8 (1), 65–70.

[B43] Zhou Y. Wang D. Zhu J. Liu Q. Fan M. X. (2005). The role of sulfur fertilizers in balanced fertilization. Proceedings of the 1st Sino German Workshop on Aspects of Sulfur Nutrition of Plants, Shenyang, China, May 2004, Vol. 283. 171–176. Agricultural Research, Bundesallee, Braunschweig, Germany: Landbauforschung Völkenrod, Federal Agricultural Center.

